# Quercetin and polycystic ovary syndrome

**DOI:** 10.3389/fphar.2022.1006678

**Published:** 2022-12-16

**Authors:** Congshun Ma, Qianru Xiang, Ge Song, Xuefei Wang

**Affiliations:** ^1^ NHC Key Laboratory of Male Reproduction and Genetics, Guangzhou, China; ^2^ Department of Reproductive Medicine Center, Guangdong Provincial Reproductive Science Institute (Guangdong Provincial Fertility Hospital), Guangzhou, China; ^3^ Guangdong Provincial Key Laboratory of Tropical Disease Research, Department of Nutrition and Food Hygiene, School of Public Health, Southern Medical University, Guangzhou, China; ^4^ Department of Obstetrics and Gynecology, Nanfang Hospital, Southern Medical University, Guangzhou, China

**Keywords:** polycystic ovary syndrome, quercetin, insulin resistance, obesity, metabolic syndrome, hyperandrogenemia, hyperandrogenism (HA)

## Abstract

Polycystic ovary syndrome (PCOS) is a reproductive endocrine disease, and results to opsomenorrhea or amenorrhea, hairy, acne, acanthosis, infertility, abortion. In the long term, PCOS may also increase the risk of endometrial cancer, diabetes, hypertension, dyslipidemia and other diseases. Till now there is no specific drug for PCOS due to the unclearness of the cause and pathogenesis, as current treatments for PCOS only target certain symptoms. Quercetin (QUR) is a flavonoid drug widely found in Chinese herbal medicines, fruits, leaves, vegetables, seeds and plants roots. Studies on other diseases have found that QUR has anti-oxidant, anti-inflammatory, anti-insulin resistance, anti-cancer and other effects. Some studies have shown that serum testosterone (T), luteinizing hormone (LH), the LH/follicule-stimulating hormone (FSH) ratio, fasting glucose, fasting insulin, HOMA-IR and lipid levels are reduced in PCOS patients with QUR treatment. However, the mechanisms of QUR in PCOS patients have not been completely elucidated. In this review, we retrospect the basic characteristics of QUR, and *in vitro s*tudies, animal experiments and clinical trials of QUR and plant extracts containing QUR in the treatment of PCOS. We also summarized the effects and mechanism of QUR in ovarian cells *in vitro* and PCOS model rats, the changes in relevant parameters after QUR administration in PCOS patients, and its potentially therapeutic applications.

## 1 Introduction

Polycystic ovary syndrome (PCOS) was first described in 1935 by Stein and Leventhal (Stein and Leventhal, 1935), which is a common polygenic endocrine disorder (Norman et al., 2007), characterized by clinical hyperandrogenism or hyperandrogenemia (HA), ovulatory dysfunction (OD), and polycystic ovary morphology (PCOM) (Rotterdam, 2004). Epidemiological surveys in recent years suggests that the global prevalence of PCOS in women was 4.4–18.6% (Ding et al., 2017), the prevalence of PCOS is 8%–13% in fertile woman (Bozdag et al., 2016) and 3%–11% in adolescent girls (Naz et al., 2019). Women with PCOS receive more infertility counseling and treatment, suffering from a lower likelihood and a longer labor time of the first delivery (Palomba, 2021). PCOS and its complications, including pregnancy-induced hypertension, pre-eclampsia, gestational diabetes mellitus, and preterm delivery (Palomba et al., 2015), significantly increase the hospitalization rate of women and bring a great economic burden to patients, families and society (Hart and Doherty, 2015). PCOS is the result of a combination of genetic and environmental factors, often accompanied by endocrine abnormalities (androgen excess exposure *in utero*, early pubarche, and seborrheic alopecia), metabolic abnormalities (obesity, metabolic syndrome, and type 2 diabetes) and reproductive dysfunction (intrauterine development disturbances, low birth weight, and infertility) (Jones and Goodarzi, 2016; Merkin et al., 2016; Deswal et al., 2020). Although the discussion on its pathogenesis has not been unified, recent researches have demonstrated that HA and insulin resistance (IR) are not only common manifestations, both may also be the core causes of PCOS (Hosseinpanah et al., 2014; Rosenfield and Ehrmann, 2016; Gu et al., 2022). The diagnosis of PCOS is most often based on the 2003 Rotterdam Criteria in clinical practice, which requires the presence of two of the following three features: OD (oligo- or anovulation), clinical/biochemical HA (hirsutism, acne, or androgenetic alopecia) and PCOM on ultrasound (an ovary with at least 20 follicles and having a diameter of 2–9 mm or a volume of at least 10 cm^3^) (Rotterdam, 2004). However, potential racial differences make the diagnostic criteria of PCOS unable to be uniform in all regions of the world (Bani Mohammad and Majdi Seghinsara, 2017; Neven et al., 2018; Joham et al., 2022), limiting the research on the pathogenesis and treatment strategies of PCOS.

Because the etiology and the specific pathogenesis have not been completely acknowledged, the PCOS treatment is still mainly based on education, counseling, and lifestyle adjustment (appropriate diets and physical activity) (Ells et al., 2018; Palomba et al., 2021). Regular healthy eating and physical activity habits benefit women with PCOS overall health and optimize their hormonal balance (Moran et al., 2020). Surgery and medication are considered as more aggressive treatments. Surgical treatment is mainly bariatric surgery, which has many risks of postoperative complications and is only suitable for extremely obese patients. The treatment drugs for PCOS are mainly divided into two categories: hormones and hypoglycemic drugs. Oral contraceptives, a combination of estrogen and progestion, have been the mainstay of effective treatment for menstrual irregularities, hirsutism, and acne in PCOS patients (Rubin et al., 2017). As the first-line treatment, oral contraceptives for long-time ingestion have been shown to have a close relationship with the occurrence of hypertension, venous thrombosis, breast tumor and other cancers (Dorchak et al., 2018). On the other hand, different diabetes drugs, such as metformin, thiazolidinediones (TZD), glucagon-like peptide-1 receptor (GLP-1R) agonists, dipeptidyl peptidase-4 (DPP-4) inhibitors, and sodium-glucose cotransporter (SGLT2) inhibitors, could be used as treatment strategies for PCOS (Javed et al., 2019; Devin et al., 2020). Although metformin is mentioned in the 2018 international evidence-based guideline for the assessment and management of PCOS (Teede et al., 2018) as the only insulin-sensitizing agent, its treatment regimen has not yet been standardized. Other diabetes drugs are controversial in treatment combinations, intake doses and target populations, still in the experimental treatment phase (Romualdi et al., 2020). Therefore, it is particularly important to select a therapy with high safety, more selectivity, and fewer adverse reactions reported. Complementary and alternative medicine (CAM) therapy is possibly a potentially effective treatment for PCOS with these advantages. There are many historical documents describing traditional medicine, which use active ingredients from plants, fungi, and microorganisms to treat various ailments. Recently, researches on CAM have made some progress in PCOS treatment, such as flavonoids, lipoic acids, inositol, coenzyme Q_10_, berberine, probiotics, melatonin, fish oil, fatty acids, vitamin D, vitamin K, carnitine, chromium, and selenium. They are found to have a certain therapeutic effect on PCOS (Zhang et al., 2021). Recent studies have highlighted the beneficial effects of flavonoids in the daily diet and have shown that flavonoid intake is effective in reducing the risk of chronic metabolic diseases (Hoseini et al., 2019). Quercetin (QUR), as a kind of flavonoid, cannot be disregarded in CAM treatment.

QUR [2-(3,4-dihydroxyphenyl)-3,5,7-trihydroxy-4-Hchromen-4-one] is one of the most abundant dietary flavonoids found in fruits and green leafy vegetables, as well as many seeds, buckwheat, nuts, flowers, barks, broccoli, olive oil, apples, onions, green tea, red grapes, red wine, dark cherries, and berries such as blueberries and cranberries. It also exists in many Chinese herbal medicine and compound Chinese herbal medicine decoction, and, as we know, that cannot be produced in the human body. Nowadays, QUR is one of the highest daily intake flavonoids (Hisanaga et al., 2016) and used as a dietary supplement and could be added to the daily diet to help with chronic diseases. Many studies demonstrated that QUR, when consumed in tolerable doses, may have beneficial biological effects, like antioxidant, anticancer, and anti-inflammatory effects (Li et al., 2016). QUR has been traditionally used to prevent or treat a variety of diseases such as cardiovascular diseases (Rodrigo et al., 2022), nervous and neurodegenerative disorders (Guo et al., 2022), diabetic nephropathy (Hu et al., 2022), cerebral ischemia (Guo et al., 2022), obesity (Yan et al., 2022), chronic inflammation (Sato and Mukai, 2020; Ortega and Jastrzebska, 2021), cancer (Parvaresh et al., 2016; Koren Carmi et al., 2020; Yang et al., 2021a; Azeem et al., 2022; Mirazimi et al., 2022; Zhu et al., 2022), and different types of bacterial and viral diseases (Nguyen and Bhattacharya, 2022). Moreover, QUR has been extensively investigated as a potential therapeutic option for PCOS patients recently (Rashidi et al., 2019; Calcaterra et al., 2021; Zheng et al., 2022a; Mirazimi et al., 2022). However, current reviews of studies on the treatment of QUR in PCOS focused on the description of its efficacy and characterizations, but few studies have explored and summarized its potential mechanism (Chen et al., 2022b).

Therefore, it is necessary to conduct a comprehensive review on the treatment of PCOS by QUR from three aspects: *in vitro* studies, animal experiments and clinical trials. This review focuses on the biomedical roles of QUR in PCOS. We retrospect *in vitro* studies of QUR on ovarian cells, clinical trials and animal experiments of QUR and plant extracts containing QUR in the treatment of PCOS, described the origin and metabolism of QUR and its pharmacokinetic features. We generalized the effects of QUR on ovarian cells *in vitro*, summarized the changes of relevant parameters in PCOS patients after QUR application, and expounded the effects of QUR on sex hormone and glucolipid metabolism in PCOS rats. Based on the existing studies on the pathogenesis of PCOS and the biomedical effects of QUR, its potential mechanism on PCOS was discussed, which is of important practical significance to clarify the occurrence and development process of PCOS and broaden the application scope of QUR and plant extracts containing QUR.

## 2 Methods

**FIGURE 1:**
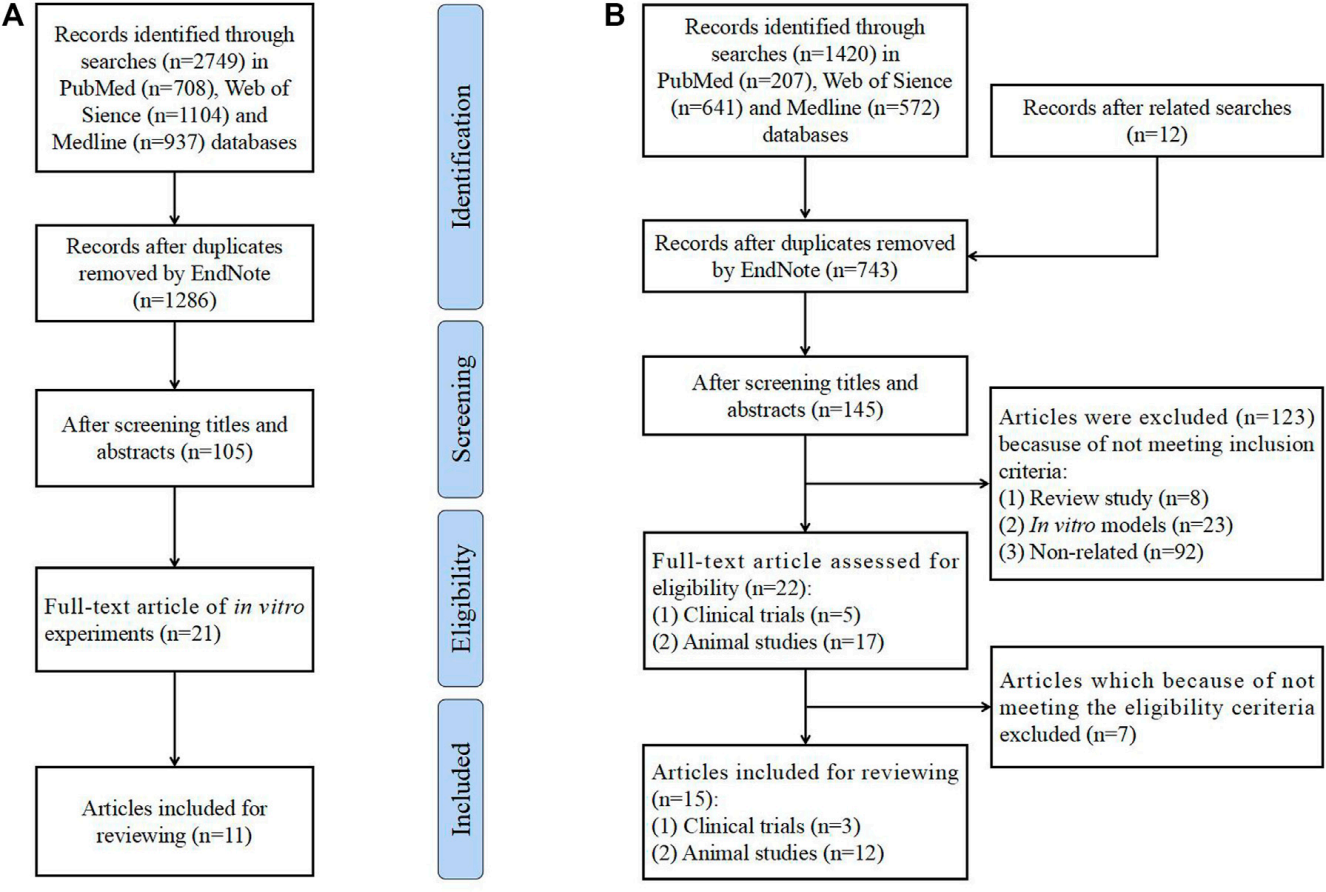
Flow diagram of the literature search and study selection process. **(A)**. Flow diagram of *in vitro* studies. **(B)**. Flow diagram of clinical trials and animal experiments.

This review searched studies published in the PubMed, Web of Science and Medline databases on the treatment of QUR in PCOS from establishment to November 2022. Terms “quercetin”, “polycystic ovary syndrome”, “insulin resistance”, “hyperandrogenemia”, “obesity”, “glucose metabolism”, “lipid metabolism”, and “chronic inflammation” have been searched. Finally, *in vitro* studies on the effect of QUR on ovarian cells have been included, as shown in Figure 1A. Search terms, “quercetin”, “polycystic ovary syndrome”, “ovulation disorders”, “insulin resistance” and “hyperandrogenemia”, are also included. For example, the query formula is “(((((polycystic ovary syndrome [Title/Abstract]) OR (PCOS [Title/Abstract])) OR (ovulation disorders [Title/Abstract])) OR (insulin resistance [Title/Abstract])) OR (hyperandrogenemia [Title/Abstract])) AND (QUR [Title/Abstract])” in the PubMed database. In addition, references and related records were reviewed. Clinical trials and animal experiments on the treatment of PCOS by QUR have been included. The flow diagram is shown in Figure 1B.

## 3 QUR and its role in ovarian cells

### 3.1 Physical features and metabolism of QUR

The molecular formula for QUR is C_15_H_10_O_7_, and its molecular weight is 302.2357 g/mol. QUR consists of a heterocyclic pyrone ring connecting two benzene rings to form a central nucleus, whose form is a hydrophilic glycoside (sugar conjugate) in herbal medicine. It is a yellow needle or yellow powder at room temperature and melts at 316.5°C. QUR is not soluble in water but soluble in organic solvents such as alcohols. When dissolved in alcohol, QUR tastes very bitter and releases pungent delays when heated to break down.

Although QUR is a foodborne nutrient compound, it is not easily absorbed directly by intestinal cells (Russo et al., 2014). In intestinal cells, QUR undergoes various enzymatic reactions by specific transferases, including methylation, hydrolysis, sulfonylation, and glucuronidation (Brito et al., 2015). These reactions are thought likely to take place under the influence of various intestinal flora, mainly *Firmicutes* and *Bacteroidetes* (Xiao and Lee, 2018). QUR is absorbed into the blood from the intestinal lumen, and then transported to liver to be metabolized as QUR-derived circulating compounds (QUR-3-glucuronide and QUR-3ʹ-sulfate) (Mirazimi et al., 2022). QUR is available in two forms: conjugated and unconjugated (3.5–5.0 and <0.33 μmol/L, respectively in the plasma) (D'Andrea, 2015). The conjugated form is more easily absorbed by intestinal cells, and its glycoside metabolites exert important biological activities (Mirazimi et al., 2022).

### 3.2 Bioavailability of QUR and its metabolites

**FIGURE 2:**
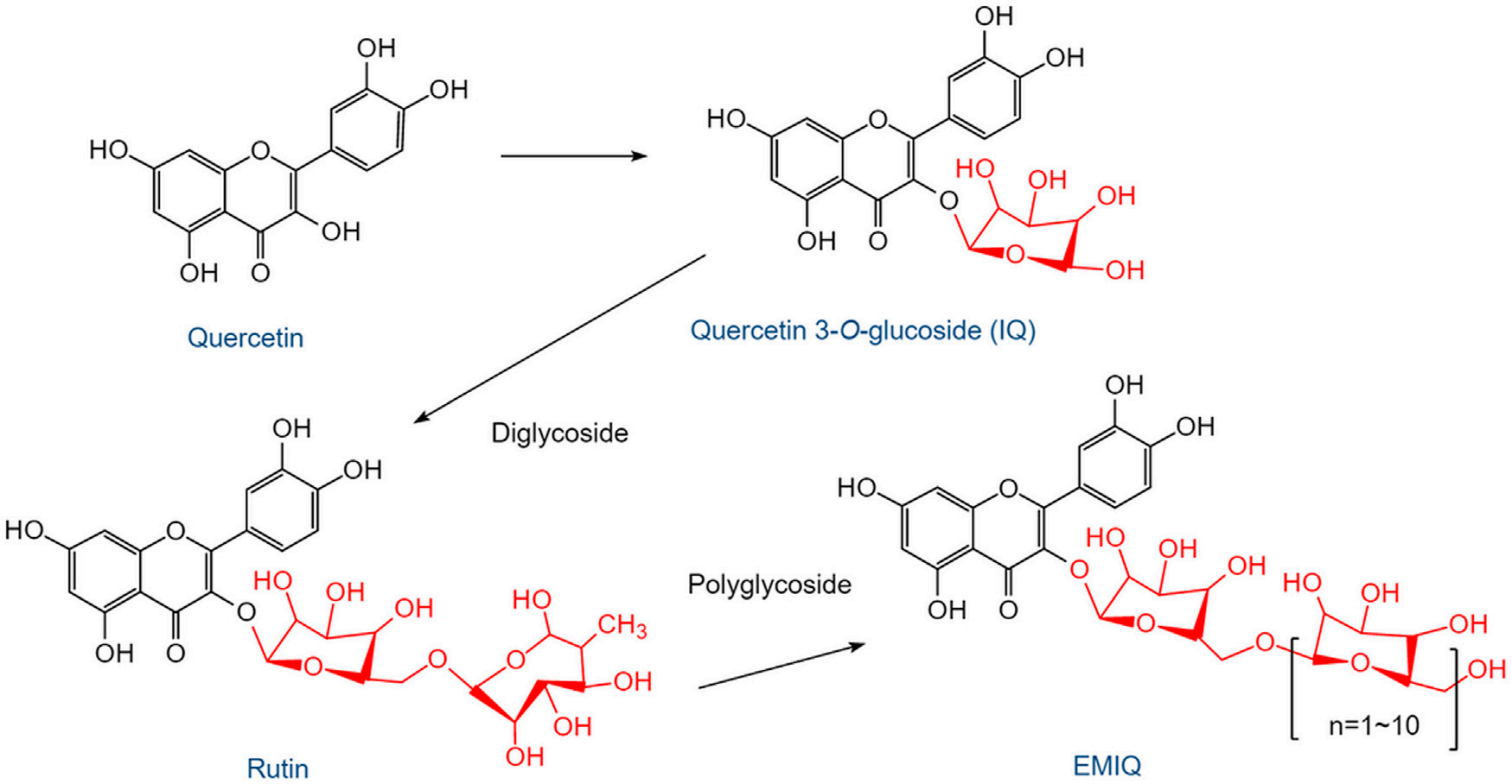
The structures and transformation process of QUR and its important glycoside metabolites. After ingesting QUR from plants, mammals absorb it under the action of microorganisms in the gut. QUR undergoes a glycosylation process to form QUR 3-*O* glycosides, the most important of which is IQ. IQ can continue to combine with glycosides to form the diglycoside QUR, also known as rutin. Rutin can also form IQ by deglycosidation. If rutin is further bound to several glycosides, EMIQ will be formed. IQ, isoquercetin; EMIQ, enzymatically modified isoquercetin.

The earliest human study of QUR reported very poor oral bioavailability (∼2%) after a single dose (Gugler et al., 1975). The absolute bioavailability of QUR in humans was estimated to be 44.8% (Walle et al., 2001). The half-lives of QUR atoms and their metabolites are in the range of 11–28 h, suggesting that continuous supplementation may lead to significant increases in plasma concentrations (Boots et al., 2008). The structures and transformation process of QUR and its important glycoside metabolites are shown in Figure 2, where the glycosylation of QUR is an important process to improve its bioavailability. After the formation of isoquercetin (IQ), it continues to combine with glycosides to form rutin, which has been proven to have various biological effects, including anti-tumor, anti-oxidation, and anti-aging (Alizadeh and Ebrahimzadeh, 2022). Continued binding of rutin to glycosides generates enzymatically modified IQ (EMIQ), which persists in the blood circulation or tissue for a long time. The anti-obesity effects of QUR and QUR 3-glycoside have been continuously concerned, and it was found that the latter showed more effectivity than the former (Lee et al., 2017). The bioavailability of QUR 3-*O* glycoside is 2.35 times higher than that of QUR (Rice-Evans et al., 1996; Lee et al., 2017). Therefore, the biological effect of QUR after oral administration by humans is the combined action of QUR and its metabolites.

### 3.3 *In vitro* studies of QUR on ovarian cells

11 *in vitro* studies (Jia et al., 2011; Capcarova et al., 2015; Tarko et al., 2018; Sirotkin et al., 2019a; Sirotkin et al., 2019b; Khadrawy et al., 2019; Kolesarova et al., 2019; Rashidi et al., 2019; Li et al., 2020; Yang et al., 2021b; Davoodian et al., 2022) have been included and are shown in Table 1. The cell origin, *in vitro* model, QUR dosage and biological response have been reviewed.

**TABLE 1:** Effects of *In vitro* studies of QUR on Ovarian Cells.

Effect	*In vitro* model	Dosage of QUR	Biological response	References
Oxidation resistance and cell apoptosis inhabitation	Cadmium-induced oxidative damage in chicken granulosa cells	1 μg/ml	↑ Bcl-2, XIAP; ↓ Bax, caspase-3, ROS	Jia et al. (2011)
	H_2_O_2_-induced oxidative damage in primary goat luteinized granulosa cells	2 μg/ml	↑Nrf2, Bcl-2; ↓ Bax, caspase-3	Li et al. (2020)
	Tunicamycin-induced ER stress in primary buffalo granulosa cells	2 μg/ml	↑ Bcl-2; ↓Bax, ROS, PERK/CHOP	Yang et al. (2022)
Oxidation and inflammation resistance	T-2 toxin-induced oxidative damage in primary porcine ovarian granulosa cells	1, 10, or 100 ng/ml	↓ ROS, GPx, SOD	Capcarova et al. (2015)
	H_2_O_2_-induced oxidative damage in primary human granulosa cells	20 μM	↑ Nrf2, Trx; ↓ ROS	Rashidi et al. (2019)
	H_2_O_2_-induced oxidative damage in primary bovine granulosa cells	10 μM	↑Nrf2; ↓ ROS	Khadrawy et al. (2019)
Cell apoptosis inhabitation	Primary breed cattle ovarian granulosa cells	1, 10, or 100 μg/ml	↓ Bax, PCNA, IGF-1	Tarko et al. (2018)
	Primary non-cycling pubertal gilts granulosa cells	10 ng/ml	↑ Bcl-2; ↓Bax, PCNA	Sirotkin et al. (2019b)
	Primary pigs and cattle ovarian cells	1 or 10 ng/ml	↓ Bax, PCNA, IGF-1	Sirotkin et al. (2019a)
	Primary porcine ovarian granulosa cells	10 μM	↓ PCNA, p53, caspase-3	Kolesarova et al. (2019)
	Primary bovine cumulus-oocyte complexes cells	2 μg/ml	↑ OCT-4, IGF2R, Bcl-2, GLUT-4; ↓ Bax, CHOP	Davoodian et al. (2022)

QUR, quercetin; ROS, reactive oxygen species; Nrf2, NF-E2-related factor 2; PERK: protein kinase R-like ER, kinase; CHOP, C/EBP-homologous protein; GPx, Glutathione peroxidase; Trx, thioredoxin; PCNA, proliferating cell nuclear antigen; IGF-1, insulin-like growth factors -1; OCT-4, Octamer-binding transcription factor 4; IGF2R, insulin like growth factor 2 receptor; GLUT-4, glucose transporter four; XIAP, X-linked inhibitor of apoptosis protein; SOD, superoxide dismutase; Bcl-2, B-cell lymphoma-2; Bax, Blc-2, associated X protein.

The collection of primary human ovarian granulosa cells was only described in one study (Rashidi et al., 2019). Hydrogen peroxide induced oxidative stress in granulosa cells, and then 20 μM QUR intervention showed that reactive oxygen species (ROS) decreased, NF-E2-related factor 2 (Nrf2) and thioredoxin (Trx) increased, thereby cell damage was repaired. Similar results were seen in granulosa cells of goats and bovines (Khadrawy et al., 2019; Li et al., 2020). In Cadmium-induced chicken granulosa cells, QUR increased X-linked inhibitor of apoptosis protein (XIAP) and decrease caspase-3 and ROS (Jia et al., 2011). QUR not only relieves cell damage by reducing oxidative stress, but also repairs cells by inhibiting apoptosis. QUR has been found to increase B-cell lymphoma-2 (Bcl-2) and decrease Blc-2 associated X protein (Bax) in ovary cells of cattle, gilts, buffalo, goat, chicken and bovine (Jia et al., 2011; Sirotkin et al., 2019b; Li et al., 2020; Yang et al., 2021b; Davoodian et al., 2022). However, it is difficult to prove that the same effect applies to humans because there is rare research on primary granulosa cells from humans. In addition, there is no PCOS model for ovarian cells, which could only be simulated by oxidative stress damage model. Oxidative stress injury is only a small part of the manifestations of PCOS and does not fully reflect the effect of quercetin on ovarian cells. Therefore, further exploration of PCOS model *in vitro* experiment is needed.

## 4 QUR studies on PCOS animal models

12 animal experiments (Shah and Patel, 2016; Wang et al., 2017; Hong et al., 2018; Jahan et al., 2018; Neisy et al., 2019; Jafari Khorchani et al., 2020; Olaniyan et al., 2020; Mihanfar et al., 2021; Zheng et al., 2022a; Hussain et al., 2022; Mahmoud et al., 2022; Younas et al., 2022) were included and are shown in Table 2. The PCOS-related animal studies involved in this review and the changes in the related indicators after QUR treatment were basically from the data letrozole induced or the dehydroepiandrosterone (DHEA) induced PCOS model. This section reviews the changes of body weight, ovarian pathology, blood biochemical parameters, related gene expression and associated protein expression in PCOS models after QUR treatment.

**TABLE 2:** Animal experiments of QUR in the treatment of PCOS.

References	Source	Model	Group (n)	Dosage	Treat time	Results of end point in the PCOS and treatment groups (mean ± SEM or mean±*SD*)								Biological response
						Body weight, g	Ovaries weight, mg	Uterus weight, mg	TC, mg/dl	TG, mg/dl	T, μU/ml	LH, μU/ml	INS, μU/ml	
Shah & Patel (2016)	Gujarat, India	SD female rats with PCOS induced by testosterone propionate (10 mg/kg/d, s.c.) for 6 weeks	PCOS (12); QUR (12)	QUR 150 mg/kg/d, p.o	5 weeks after being induced	NM	*99.00 ± 3.86; 75.60 ± 6.86	*296.7 ± 5.11; 263.0 ± 8.60	*208.9 ± 8.43; 141.7 ± 3.10	*137.2 ± 19.19; 74.6 ± 12.9	*0.65 ± 0.02; 0.29 ± 0.02	*20.60 ± 0.28; 15.10 ± 0.36	*12.46 ± 0.30; 10.00 ± 0.28	↓ Phosphatidylinositide 3-kinase, CYP17A1
Wang et al. (2017)	Shanghai, China	Wistar female rats with PCOS induced by DHEA (60 mg/kg/d, s.c.) for 20 days	PCOS (12); QUR (12)	QUR 100 mg/kg/d, p.o	5 weeks after being induced	*193.53 ± 3.61; 185.54 ± 4.64	89.95 ± 8.81; 93.09 ± 17.61	NM	NM	NM	NM	NM	NM	↓ INS, IL-1β, IL-6, TNFα, TLR/NF-κB
Hong et al. (2018)	Nanjing, China	SD female rats with PCOS induced by letrozole (1 mg/kg/d, s.c.) for 21 days	PCOS (6); QUR (6)	QUR 25 mg/kg/d, p.o	21 days while being induced	*117.56 ± *11.24*; 95.24 ± *9.87*	NM	NM	NM	NM	*0.36 ± *0.03*; 0.31 ± *0.02*	NM	NM	↓ SOD, CAT, GPx
Jahan et al. (2018)	Islamabad, Pakistan	SD female rats with PCOS induced by letrozole (1 mg/kg/d, s.c.) for 21 days	PCOS (6); QUR (6)	QUR 30 mg/kg/d, p.o	21 days while being induced	223.16 ± 3.85; 199.28 ± 2.75	*104.60 ± 2.20; 78.20 ± 2.00	NM	*63.96 ± 0.69; 55.61 ± 1.26	*68.06 ± 1.42; 56.07 ± 0.78	*1.69 ± 0.17; 0.78 ± 0.14	NM	NM	NM
Neisy et al. (2019)	Shiraz, Iran	SD female rats with PCOS induced by DHEA (60 mg/kg/d, s.c.) for 21 days	PCOS (7); QUR (7)	QUR 15 mg/kg/d, p.o	30 days after being induced	268.78 ± 4.25; 241.63 ± 3.95	NM	NM	NM	NM	NM	NM	30.12 ± 3.65; 18.75 ± 5.65	↑HK, GK; ↓ ERα, GLUT4
Jafari Khorchani et al. (2020)	Shiraz, Iran	SD female rats with PCOS induced by DHEA (60 mg/kg/d, s.c.) for 21 days	PCOS (7); QUR (7)	QUR 15 mg/kg/d, p.o	30 days after being induced	NM	NM	NM	NM	NM	NM	NM	NM	↑adipoR1, nesfatin-1
Olaniyan et al. (2020)	Edo State, Nigeria	Wistar female rats with PCOS induced by DHEA (60 mg/kg/d, s.c.) for 21 days	PCOS (7); QUR (7)	QUR 100 mg/kg/d, p.o	15 days after being induced	NM	NM	NM	NM	NM	NM	NM	NM	↑MDA, Bcl2, E-Cadherin; ↓ Bax
Mihanfar et al. (2021)	Urmia, Iran	Wistar female rats with PCOS induced by letrozole (1 mg/kg/d, s.c.) for 21 days	PCOS (6); QUR (6)	QUR 100 mg/kg/d, p.o	30 days after being induced	207.76 ± 5.57; 199.40 ± 5.27	89.93 ± *16.65*; 82.28 ± *19.35*	*184.14 ± *45.58*; 335.29 ± *93.0*	*83.52 ± *7.68*; 58.08 ± *2.40*	*144.8 ± *9.24*; 66.68 ± *4.83*	*0.36 ± *0.60*; 0.09 ± *0.01*	NM	*26.21 ± *3.44*; 16.17 ± *2.92*	↑AMPK, SIRT-1
Zheng et al. (2022a)	Guangzhou, China	SD female rats with PCOS induced by DHEA (60 mg/kg/d, s.c.) for 20 days	PCOS (10); QUR (10)	QUR 100 mg/kg/d, p.o	28 days after being induced	NM	NM	NM	NM	NM	0.782 ± *0.05*; 0.633 ± *0.04*	35.25 ± *3.98*; 27.86 ± *4.17*	NM	↓Bax, IL-1β, IL-6, TNF-α, AR, CNP/NPR2; ↑ Bcl2
Mahmoud et al. (2022)	Cairo, Egypt	Wistar female rats with PCOS induced by DHEA (60 mg/kg/d, s.c.) for 41 days	PCOS (7); QUR (7)	QUR 25 mg/kg/d, p.o	4 weeks after being induced	*174.65 ± 4.96; 152.77 ± 5.68	*100.00 ± 8.00; 140.00 ± 10.00	NM	NM	NM	*0.36 ± 0.04; 0.18 ± 0.01	*25.14 ± 2.68; 12.89 ± 3.68	NM	↓Bax,; ↑ Bcl2
Younas et al. (2022)	Faisalabad, Pakistan	Wistar albino female rats with PCOS induced by letrozole (1 mg/kg/d, s.c.) for 7 weeks	PCOS (5); *Fagonia indica* (5)	Extract of *Fagonia indica* (QUR 0.904 mg/g) 500 mg/kg/d, p.o	7 weeks after being induced	164.58 ± 3.45; 176.48 ± 6.88	NM	NM	*104.00 ± 2.56; 74.68 ± 1.68	*91.52 ± 0.98; 86.25 ± 1.77	*3.96 ± 0.02; 2.21 ± 0.95	4.56 ± 0.02; 4.01 ± 0.06	16.32 ± 0.02; 14.89 ± 0.68	↓DPPH, CAT, GSH, SOD
Hussain et al. (2022)	Faisalabad, Pakistan	Swiss albino female rats with PCOS induced by letrozole (1 mg/kg/d, s.c.) for 4 weeks	PCOS (5); Bitter Melon (5)	Extract of Bitter Melon (QUR 0.765 mg/g) 500 mg/kg/d, p.o	4 weeks after being induced	172.35 ± 7.25; 176.84 ± 3.65	NM	NM	NM	NM	*0.32 ± 0.02; 0.16 ± 0.03 ng/ml	*17.25 ± 2.35; 13.11 ± 2.94	*13.04 ± 1.89; 9.44 ± 1.91	↓DPPH, CAT, ROS, SOD

QUR, quercetin; PCOS, polycystic ovary syndrome; SD, Sprague–Dawley; *SD*, standard deviation; SEM, standard error of mean; DHEA, dehydroepiandrosterone; ROS, reactive oxygen species; SOD, superoxide dismutase; GPx, Glutathione peroxidase; GSH, glutathione; CAT, catalase; DPPH, 2,2-Diphenyl-1-picrylhydrazyl; BMI, body mass index; T, testosterone; TC, cholesterol; TG, triglycerid; SHBG, sex hormone-binding globulin; LH, luteinizing hormone; INS, insulin; HOMA-IR, homeostasis model assessment insulin resistance; s.c., subcutaneous injection; p.o., peros; NM, not mentioned; PI3K, Phosphatidylinositide 3-kinases; CYP17A1, cytochrome P450 family 17 subfamily A member one; NF-κB, nuclearfactorkappa-B; CNP, C-type natriuretic peptide; NPR2, natriuretic peptide receptor two; IL-1β, interleukin-1β; TNF-α, tumor necrosis factor-α; IL-6, interleukin-6; AR, androgen receptor; MDA, malondialdehyde; HK, hexokinase; GK, glucokinase; ERα, estrogen receptor α; GLUT4, glucose transporter four; TLR, Toll-like receptors; Bcl-2, B-cell lymphoma-2; Bax, Blc-2, associated X protein; *, *p* < 0.05. The values in the table are converted from data of diagrams or tables in the reference literature.

### 4.1 QUR effects animal weight, estrous cycle, ovary and uterus

Pre vious studies have been confirmed QUR’s antioxidant and anti-insulin resistance effectives in diabetes and metabolic syndrome (Guo et al., 2013; Li et al., 2013; Saleem and Rizvi, 2017; Chen and Pang, 2021; Hosseini et al., 2021; Tan et al., 2021; Chen et al., 2022a; Jiang et al., 2022; Yan et al., 2022). It is well known that weight gain is considered one of the most important clinical features of PCOS (Kauffman et al., 2008; Lewandowski et al., 2011; Szosland et al., 2019). A study demonstrated a 24% increase in average body weight in letrozole-induced PCOS rats compared to the control group or the PCOS-QUR group at the end of the experiment (Jahan et al., 2018). Another study on letrozole-induced PCOS rats also showed that at the end time, the body weight of the PCOS group was higher than that of the control group or the PCOS-QUR group (Mihanfar et al., 2021). Body weight gain was also found in DHEA-induced PCOS rats compared to the control group, while weight loss was shown after QUR treatment (Neisy et al., 2019). It is suggested that weight gain in PCOS animals is not only caused by obesity, but may also be related to the changes of ovarian or uterine weight (Jahan et al., 2018; Mihanfar et al., 2021). The ovarian index of the DHEA-induced PCOS model group significantly increased in contrast to the control group (Jahan et al., 2018; Zheng et al., 2022a), while uterine weight was significantly reduced in letrozole-induced PCOS rats (Mihanfar et al., 2021). The diameter and weight of the ovary were 68.16 ± 3.12 mm and 5.23 ± 0.11 mg respectively in letrozole-induced PCOS rats *versus* the control group 28.50 ± 1.08 mm and 3.6 ± 0.14 mg, while those in the PCOS-QUR group were 33.85 ± 0.55 mm and 3.91 ± 0.10 mg (Jahan et al., 2018). Naisy’s study is consistent with the above results (Neisy et al., 2019), suggesting that QUR could reduce body and ovarian weight in PCOS rats, which is beneficial to alleviating obesity. Changes in body weight of PCOS animals under QUR treatment are thought to be jointly determined by the body weight of adipose tissue and organs, especially sexual organs.

The estrous cycle of the PCOS model group rats disordered and continued to be in estrus, which has been observed in many studies (Wang et al., 2017; Jahan et al., 2018; Nallathambi and Bhargavan, 2019; Pourteymour Fard Tabrizi et al., 2020; Mihanfar et al., 2021; Zheng et al., 2022a). The estrous cycle can be researched on the histology of the ovaries and uterine in addition to animal behavior. The main pathological changes of endometrial glands in the uterus of the letrozole-induced PCOS model rat group were the endometrial gland hyperplasia by H&E staining, while the ovarian sections showed a thin granular layer and cystic follicles (Zheng et al., 2022a). Studies have compared the QUR with the metformin and found that they have the equal effects in ovarian pathology in PCOS rats (Mahmoud et al., 2022). QUR group had decreased cystic follicles and significantly increased corpus luteum and normal follicles in DHEA-induced PCOS rats (Jahan et al., 2018). Several studies have demonstrated that QUR was effective in normalizing the irregular estrous cycle (Jahan et al., 2018; Zhang et al., 2021; Mahmoud et al., 2022), primarily by increasing the number of preantral, antral, and preovulatory follicles and corpora lutea counts, as well as decreasing atretic follicle counts and eliminating the formation of cysts in the PCOS rats (Olaniyan et al., 2020; Mahmoud et al., 2022). However, due to the limited number of studies, more and larger animal experiments are needed.

### 4.2 QUR effects on steroid hormones

One of the main clinical features of PCOS patients is HA, manifested as elevated serum testosterone (T), free T, LH, LH/follicule-stimulating hormone (FSH) ratio, and SHBG levels (Rosenfield and Ehrmann, 2016; Saleem and Rizvi, 2017; Zhang et al., 2019; Zhang et al., 2020; Ding et al., 2021); similar changes were observed in steroid levels in rats with DHEA or letrozole-induced PCOS (Jahan et al., 2018; Poojary et al., 2022). QUR could alter the levels of sex hormones in PCOS rats, especially reducing active androgens. A study showed that the serum T, estradiol (E2), LH, LH/FSH were reduced and the serum FSH was increased after given 2 ml QUR solution (100 mg/kg) by gavage daily for 28 days in DHEA-induced PCOS rats, whose effects were similar to that of DHEA-induced PCOS rats after metformin gavage in another study (Zheng et al., 2022a). After being orally administered with QUR (25 mg/kg) for 28 days, the results of hormone levels in DHEA-induced PCOS rats were similar to the previous study, that the free T levels were reduced in the QUR and metformin groups (Mahmoud et al., 2022). The serum free T in PCOS rats was also decreased significantly by using the QUR-rich extract of Bitter Melon and Fagonia indica (Hussain et al., 2022; Younas et al., 2022).

A significant decrease in the levels of progesterone and E2 and an increase in serum T was observed in letrozole-induced PCOS rats (Jahan et al., 2018; Mihanfar et al., 2021). A recent study also found that serum LH and free T levels were higher in the E2-induced PCOS rats, whereas the E2 level and ovarian aromatase protein content were significantly lower than those values recorded in the control group. Additionally, the LH/FSH ratio was significantly higher in the DHEA-induced PCOS rats, and the E2/free T ratio was reduced in the DHEA group than the control group (Mahmoud et al., 2022). Serum FSH levels in the PCOS group compared to the control group have been disputed in different studies, including slightly elevated (Saleem and Rizvi, 2017; Ding et al., 2021), no difference (Mahmoud et al., 2022), and decreased (Zheng et al., 2022a). This phenomenon may be related to the different induced model and measurement methods.

It is found that using different doses of QUR (ranged from 15 to 100 mg/kg) reduced LH, the LH/FSH ratio, T, and free T and SHBG levels equally well in letrozole-induced PCOS rats (Jahan et al., 2018; Mihanfar et al., 2021; Zheng et al., 2022a; Mahmoud et al., 2022). Those effects on optimizing steroid hormone levels in the PCOS model were close to that of using the standardized metformin regimen (Jahan et al., 2018; Mihanfar et al., 2021; Zheng et al., 2022a; Mahmoud et al., 2022). From those studies of the PCOS rat model, it could be seen that the PCOS model rats were accompanied by varying degrees of HA and alterations in related parameters, and the use of different doses of QUR may normalize the abnormal changes caused by the PCOS inducer.

### 4.3 QUR effects on parameters of glucose lipid metabolism and hepatic enzymes

The role of abnormal glucose and lipid metabolism in the disease development has been confirmed by multiple studies, where HA and IR are key links (Rosenfield and Ehrmann, 2016; Saleem and Rizvi, 2017; Poojary et al., 2022). The successful replication of PCOS animal models with only ultra-physiological doses of high-glucose food perfusion indirectly illustrates the important role of insulin regulation and glucose metabolism in the pathogenesis of PCOS (Noroozzadeh et al., 2017). Therefore, plenty of studies on the pathological mechanisms and medicine effects of PCOS have focused on insulin resistance and glucose lipid metabolism (Hosseinpanah et al., 2014; Saleem and Rizvi, 2017; Chen and Pang, 2021). A study of DHEA-induced PCOS rats found that the fasting blood glucose (FBG), fasting insulin (FIS), Homeostasismodel Assessment (HOMA) index [HOMA-IR = FBG (mmol/L) × FIS (mU/L) ÷ 22.5] (Aleshin et al., 1998), and insulin levels at 30, 60, and 120 min post-meal in the DHEA-induced PCOS group were all significantly higher compared with the control group, and 57.1% of PCOS model rats were diagnosed with IR (Wang et al., 2017). At the same time, PCOS rats may be accompanied by abnormal lipid metabolism, hepatic and renal functions. The main serum lipid profile parameters such as the levels of cholesterol, triglycerides (TG), and low-density lipoprotein-cholesterol (LDL-C) were higher whereas the levels of high density lipoprotein-cholesterol (HDL-C) were reduced in letrozole-induced PCOS rats than control group (Mihanfar et al., 2021). The levels of serum alanine aminotransferase (ALT), aspartate aminotransferase (AST), and lactate dehydrogenase (LDH) in the DHEA-induced PCOS model group were significantly higher than those in the control group (*p* < 0.05) (Mahmoud et al., 2022). Neisy et al. also analyzed the changes of liver metabolic enzymes in PCOS rats, which showed that hepatic glucokinase (GK) activity was reduced and hexokinase (HK) activity was increased in DHEA-induced PCOS rats (Neisy et al., 2019).

Since QUR has a potential anti-insulin resistance effects, whether QUR affects blood glucose, blood lipid and insulin metabolism in PCOS animals is also the key points of studies. Previous studies found that QUR (daily dose range from 15 to 100 mg/kg/d) for 28–30 days (Wang et al., 2017; Jahan et al., 2018; Neisy et al., 2019; Mihanfar et al., 2021; Zheng et al., 2022a) could significantly reduce blood glucose, serum insulin, HOMA-IR, cholesterol, TG, and LDL-C levels, and elevate HDL-C levels in letrozole- or DHEA-induced PCOS rats. The reduced ALT, AST and LDH levels were observed in PCOS rats with QUR treatment in contrast to the PCOS group, when monitoring rat liver and kidney function (Mahmoud et al., 2022). Another study found that hepatic HK and GK levels were significantly increased in PCOS rats after QUR administration compared with the PCOS model group (Neisy et al., 2019). Dyslipidemia, dysglycemia, and IR status cause by PCOS were successfully optimized by QUR administration, which has similar use value to the standard positive treatment drug metformin. And these studies showed no significant differences between the effects of IR in the optimized PCOS model and those in the PCOS rats treatment with metformin (Wang et al., 2017; Jahan et al., 2018; Zheng et al., 2022b).

### 4.4 Proposed mechanisms

**FIGURE 3:**
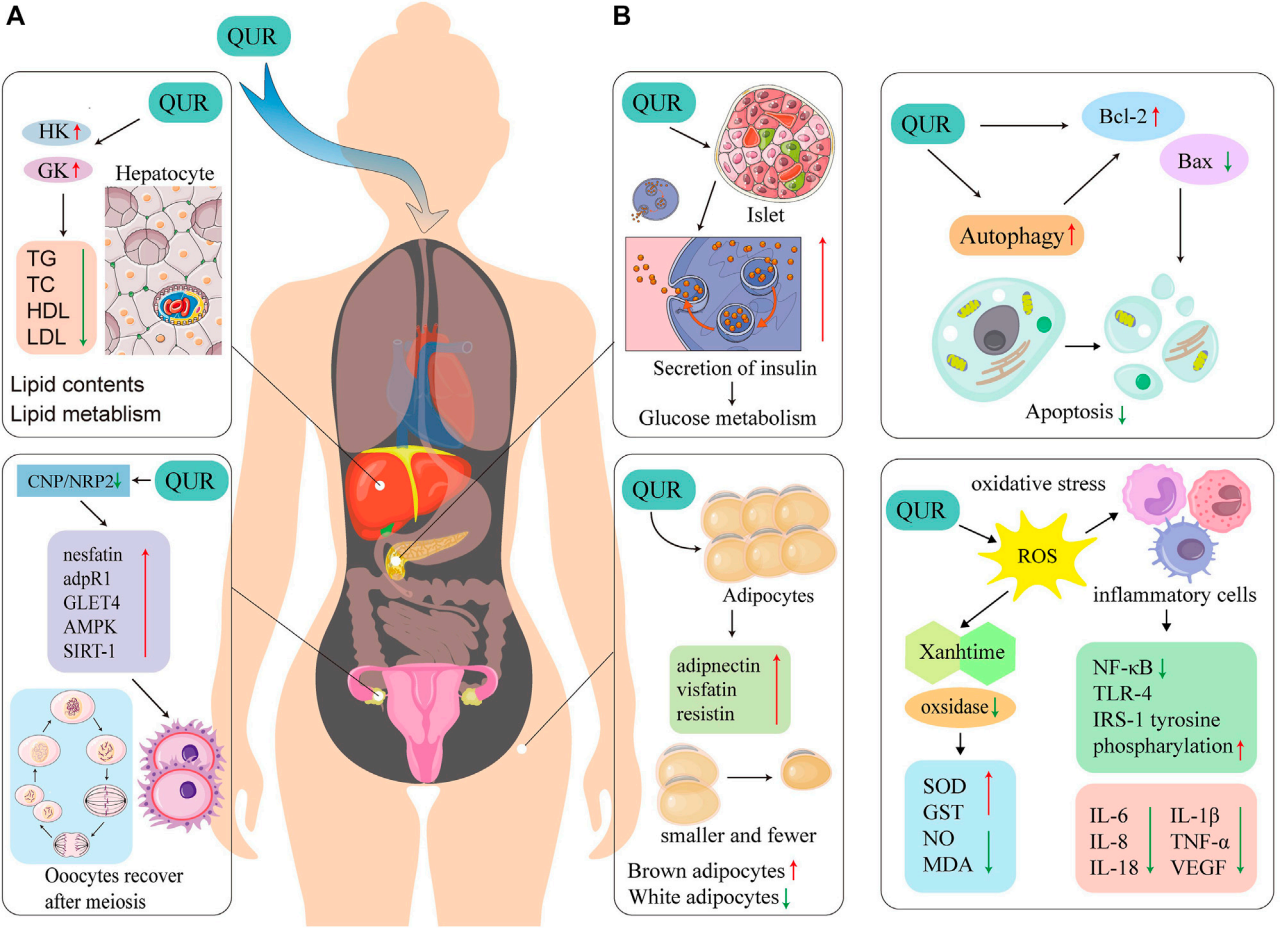
Proposed possible mechanisms of QUR treatment on PCOS. **(A)**. The possible mechanism of QUR’s role to treat PCOS in related organs. QUR can act on the pancreas to improve IR, and can act on the liver to increase HK and GK. QUR can promote the expression of nesfatin, adpR1, GLET4, AMPK and SIRT-1 in the ovary under the combined action of CNP/NRP2, thereby making the oocyte Cells repair rapidly after meiosis. QUR can act on adipose tissue to increase the secretion of adiponectin, visfatin and resistin. **(B)**. QUR can inhibit cell apoptosis and improves oxidative stress. QUR can increase the expression of Bcl-2 in the target organ, decrease the expression of Bax, to inhibit cell apoptosis. QUR can inhibit the secretion of pro-inflammatory factors by inflammatory cells, promote the secretion of anti-inflammatory factors by inflammatory cells, making IL-6, IL-8, IL-18, IL-1β, TNF-α and VEGF expression decrease through NF-κB, TLR-4, IRS-1 tyrosine and phosphorylation. QUR can inhibit oxidative stress and reduce xanthine-related oxidase, thereby increasing SOD, GST, NO, and MDA. QUR, quercetin; PCOS, polycystic ovary syndrome; IL-6, interleukin-6; IL-8, interleukin-8; IL-18, interleukin-18; IL-1β, interleukin-1β; TNF-α, tumor necrosis factor-α; VEGF, vascular endothelial growth factor; NF-κB, nuclear factor kappa-B; TLR-4, toll-like receptor 4; IRS-1, insulin receptor substrate-1; SOD, superoxide dismutase; GST, glutathione S-transferase; MDA, malondialdehyde; Bcl-2, B-cell lymphoma-2; Bax, Blc-2 associated X protein; adpR1, adipoR1; GLET4, glucose transporters; AMPK, adenosine 5‘-monophosphate-activated protein kinase; SIRT-1, sirtuin 1; GK, glucokinase; HK, hexokinase.

The potential mechanism of QUR’s effects on PCOS is shown in Figure 3.

#### 4.4.1 Anti-inflammatory effects and NF-κB inhibition

Previous animal studies showed that C-reactive protein (CRP), white blood cell count (WBC), leukocyte factor 18 (IL-18), leukocyte factor 6 (IL-6), leukocyte factor 1 (IL-1β), monocyte chemotaxis protein-1 (MCP-1), tumor necrosis factor α (TNF-α) and macrophage inflammation protein-lα (MIP-lα) levels were significantly elevated in PCOS rats. Similar abnormalities in these parameters were also demonstrated in the PCOS group compared to the control group after adjustment for patient age and body mass index (BMI) (Cirillo et al., 2019; Bannigida et al., 2020; Rudnicka et al., 2020; Wu et al., 2021).

A previous study has confirmed the anti-inflammatory effect of QUR (Azeem et al., 2022). The study has investigated the effect of QUR on the expression of Nuclear Factor Kappa B (NF-κB) and the changes of these inflammatory factors in DHEA-induced PCOS rats, and found that the levels of inflammatory cytokines contained IL-1b, IL-6, and TNFα were reduced with QUR treatment in DHEA-induced PCOS rats, and the phosphorylation of insulin receptor substrate-1 (IRS-1) tyrosine phosphorylation was significantly reduced, while the expression levels of TNF-α and NF-kBp65 were higher in the ovarian of PCOS rats than the control group, and the nuclear translocation of NF-κBp65 were significantly inhibited after QUR treatment. The elevated messenger RNA (mRNA) levels of p22phox, oxidized low density lipoprotein (OX-LDL), and Toll-like receptor 4 (TLR-4) in the PCOS rats were also significantly decreased in QUR group. Therefore, it was inferred that the mechanism of QUR may be related to the inhibition of the TLR/NF-κB signaling pathway and the improvement of the inflammatory microenvironment in the ovarian tissues of PCOS rats (Wang et al., 2017). Another research also showed a significant increase in TNF-α and vascular endothelial growth factor (VEGF) levels in DHEA-induced rats compared with the control group. However, QUR significantly reduced the levels of inflammatory factors in PCOS rats (Olaniyan et al., 2020).

#### 4.4.2 Improvement of the oxidative stress status

Previous studies have found that the levels of oxidative stress markers in follicle fluid such as ROS, total antioxidant capacity (TAC), and 8-isoprostantin (8-IP) were significantly higher in PCOS patients than in those without PCOS (Mohammadi, 2019; Ostadmohammadi et al., 2019; Gongadashetti et al., 2021). QUR inhibits the activity of xanthine oxidase, thereby alleviating oxidative damage during injury, ischemia and oxidative stress (Jafari Khorchani et al., 2020; Rashidi et al., 2021; Rodrigo et al., 2022). One study has analyzed the changes of antioxidant enzymes and the effects of QUR in DHEA-induced PCOS rats, which showed that superoxide dismutase (SOD), catalase, glutathione-S-transferase (GST), reduced glutathione levels are significantly decreased in PCOS rats, but the levels of these antioxidant enzymes were significantly increased in PCOS rats with QUR treatment (Olaniyan et al., 2020). A study from China also focused on the changes in oxidative stress-related markers in DHEA-induced PCOS rats and the effects of QUR on the expression levels of these markers, which found that serum SOD concentration decreased and malondialdehyde (MDA) concentration increased in DHEA-induced PCOS rats, while serum SOD concentration elevated, nitric oxide (NO) and MDA decreased with QUR administration in PCOS rats (Yang et al., 2021b).

More researches are required to answer whether QUR extenuates PCOS-related symptoms and improves parameters associated with anti-oxidative stress. The specific mechanism of anti-oxidative stress needs further study.

#### 4.4.3 Anti-apoptotic effects

Previous studies found that PCOS could be associated with aberrant expression of pro-and anti-apoptotic (Tan et al., 2010; Salehi et al., 2017; Olaniyan et al., 2020). It was known that B-cell lymphoma-2 (Bcl-2) is an oncogene-derived protein that provides negative control in pathways of the cellular suicide mechanisms. Blc-2 Associated X protein (Bax) is a Bcl-2 orthologue that promotes cell death by competing with Bcl-2. A study in Egypt showed that the expression levels of Bax were significantly increased, the Bcl-2 expression levels was decreased in the DHEA-induced PCOS rats compared with the control group. At the same time, the Bax/Bcl-2 ratio was significantly increased in the DHEA group (Mahmoud et al., 2022). This study also analyzed the changes of Bax and Bcl-2 expression in PCOS rats with QUR administration, showing that the Bax expression level was significantly decreased and the Bcl-2 expression level were increased after QUR treatment, which inferred that changes in these apoptosis-related proteins after QUR administration alleviated HA in PCOS rats, ultimately increasing E2 concentration, and ovarian aromatase protein content, and leading to folliculogenesis and atresia decrease (Mahmoud et al., 2022). Another study showed that Bax expression level was higher in preantral and antral follicles of the DHEA-induced rats, and decreased in preantral and antral follicles after QUR treatment with slightly increased Bcl-2 expression (Olaniyan et al., 2020). A recent study found that the average optical density (AOD) of Bcl-2 in ovaries of DHEA-induced rats was significantly decreased, accompanied by a significant increase in the AOD of Bax in ovaries of model rats (Zheng et al., 2022b). However, the AOD of Bcl-2 in the ovary was significantly increased and the AOD of Bax was significantly reduced with QUR treatment when compared with the PCOS model group of rats (Zheng et al., 2022b). Of course, whether QUR affects the expression of apoptosis-related genes and proteins such as Bcl-2 and Bax were still lacking, and whether QUR has a similar role in PCOS patients’ needs to design reasonable clinical studies.

#### 4.4.4 Down-regulating the expression of CNP/NPR2

It is known that the C-type natriuretic peptide (CNP) and its specific receptor natriuretic peptide receptor 2 (NPR2) are key signaling substances that suppresses the oocyte recovery from meiosis. And under the action of FSH, CNP binds to NPR2 to produce cyclic guanosine monophosphate (cGMP), inhibiting Phosphodiesterase 3 A (PDE3A) activity, and maintaining high levels of cyclic adenosine monophosphate (cAMP) in the oocyte cytoplasm, thus blocking oocyte meiosis. Before ovulation, peak LH could down-regulate both CNP expression and NPR2 activity, reduce both cGMP and cAMP levels, and promote the recovery of meiosis and ovulation (Zhang et al., 2010; Kiyosu et al., 2012; Tsuji et al., 2012; Barclay et al., 2015). It was found that CNP/NPR2 was abnormally expressed in DHEA-induced PCOS rats, and the trend of androgen expression in the model rats was similar to that of CNP/NPR2, suggesting that a significant increase in CNP/NPR2 led to regularity of ovulation. The expression of CNP/NPR2 was also found to be down-regulated in QUR-treated PCOS rats, inferring that QUR may affect the binding of CNP and NPR2 gene promoter-specific sequences, and regulate the transcription of CNP and NPR2 genes as well as CNP/NPR2 genes, thereby restoring ovulation and alleviating PCOS (Zheng et al., 2022b).

#### 4.4.5 Anti-insulin resistance effects

IR plays an important role in PCOS, so drugs that potentially improve the state of IR are often used for the treatment of PCOS patients, especially in PCOS patients with IR. Relevant data from previous studies showed that QUR could effectively reduce FBG, serum insulin, HOMA-IR, and blood lipid levels in PCOS model rats (Hosseinpanah et al., 2014; Saleem and Rizvi, 2017; Chen and Pang, 2021). The same phenomenon occurred in insulin-resistant PCOS patients, suggesting that QUR may be involved in the regulation of IR state.

It is well known that nesfatin-1 and adiponectin receptor 1 (adpR1) are considered as influencing factors of type 2 diabetes, obesity and PCOS. Several studies have shown that anomalies in adiponectin release and disruption in the expression of adpR1 and nesfatin-1 were associated with disorders such as HA, IR and PCOS (Ademoglu et al., 2014; Jafari Khorchani et al., 2020). The expressions of nesfatin-1 and adpR1 genes in the ovarian tissue of PCOS rats were decreased compared with PCOS rats with QUR treatment. Furthermore, it is inferred that QUR may alleviate the symptoms of obesity and infertility related to PCOS through the action of phytoestrogen and the effect of mimicking estrogen (Jafari Khorchani et al., 2020).

Another study was also focused on the effects of QUR on IR and the related mechanisms, showing that hepatic GK activity was reduced and HK activity was increased in DHEA-induced PCOS rats, and the expression of uterine estrogen receptor α (ERα) and glucose transporters (GLUT4) were significantly decreased in PCOS rats. After intragastric administration of QUR in PCOS rats, the activities of GK and HK were significantly increased, while the expressions of uterine ERα and GLUT4 were significantly increased. Therefore, it is indicated that QUR could significantly release PCOS-related IR and induce uterine GLUT4 and ERα gene expression, potentially becoming a reliable drug for the treatment of PCOS combined with IR (Neisy et al., 2019).

Previous studies have revealed that the activation of Amp-activated protein kinase (AMPK) and Sirtuin 1 (SIRT-1) is an important trigger of PCOS, while recent studies focus on the effect of QUR on AMPK and SIRT1 activation. It is showed that the expression levels of AMPK and SIRT-1 in ovarian tissue were upregulated in the letrozole-induced PCOS rats with QUR treatment. QUR also reversed the PCOS-induced alteration, and increased the levels of adiponectin, visfatin, and resistin in adipose, inferring that QUR may be a potential cause for active therapeutic effects in PCOS by regulating adipose tissue hormone release and energy balance through the expression levels involved in the AMPK and SIRT-1 axes (Mihanfar et al., 2021).

### 4.5 Limitations of animal experiments on PCOS

Because of the ethical limitations and complexity of human researches, animal models are crucial resources for understanding the features of PCOS and the effects of potential treatment methods on PCOS. At present, a natural PCOS animal has not been found. In order to conduct PCOS-related animal researches, the use of pharmacological or lifestyle interventions to promote animal models of PCOS is required. Mostly there is no clear consensus on the use of appropriate animal models and the selection of appropriate PCOS inducers. Rats and mice are usually used as experimental animals. Letrozole, dehydroepiandrosterone (DHEA), dihydrotestis, testosterone propionate, estradiol valerate, and mifepristone have been used to induce the PCOS model (Noroozzadeh et al., 2017), in which letrozole and DHEA are the most commonly used inducers (Jahan et al., 2018; Poojary et al., 2022). It is still an important research direction of PCOS animal experiments to find the animal model which is consistent with the pathogenesis of PCOS in human.

## 5 QUR for PCOS patients

Recently, researches related to QUR have become a hot spot. Since 2016, as a natural antioxidant and metabolic regulator, QUR has received increasing attention from researchers, especially for its potential effects and therapeutic value in PCOS (Shah and Patel, 2016). The limited researches mostly focus on animal experiments, and studies contained about PCOS patients are non-etheless very rarely. Four clinical trials (Rezvan et al., 2017; Khorshidi et al., 2018; Rezvan et al., 2018; Vaez et al., 2022) were included, two of which belong to a same study, as shown in Table 3. Furthermore, the U.S. National Library of Medicine (clinicaltrials.gov), a clinical trial (the U.S. National Library of Medicine; *clinicaltrials. gov*; NCT03493984) at the University of Louisville investigating the effects of exosomes from QUR-rich ginger and aloe vera on PCOS patients has not yet reported any results. These studies were summarized, and the effects of QUR on PCOS patients were discussed.

**TABLE 3:** Clinical trials of QUR in the treatment of PCOS.

References	Source	Inclusion criteria	Group (n)	Dosage	Time	Age, mean ± SD	Results of end point in the QUR and PCOS groups (mean ± SD)						
							BMI, kg/m^2^	T, ng/ml	SHBG, mmol/L	LH, mIU/ml	FSH, mIU/ml	HOMA-IR	INS, μU/ml
Rezvan et al. (2018), Rezvan et al. (2017)	Tehran, Iran	PCOS	QUR (42); Placebo (42)	QUR 1000 mg/d, p.o	12 weeks	29.45 ± 4.09; 30.00 ± 5.44	29.19 ± 3.73; 28.59 ± 4.04	*0.71 ± 0.15; 0.76 ± 0.12	41.36 ± 2.74; 35.54 ± 2.08	8.29 ± 2.99; 8.83 ± 2.00	NM	*1.88 ± 0.56; 2.17 ± 0.69	*8.45 ± 2.23; 9.81 ± 2.62
Khorshidi et al. (2018)	Tehran, Iran	PCOS with obesity or overweight	QUR (39); Placebo (39)	QUR 1000 mg/d, p.o	12 weeks	29.50 ± 4.20; 30.00 ± 5.5	29.5 ± 3.7; 28.6 ± 4.1	0.72 ± 0.15; 0.76 ± 0.12	40.8 ± 17.8; 35.4 ± 13.3	8.05 ± 2.88; 8.77 ± 1.99	NM	4 ± 1.5; 3.8 ± 1.4	18.1 ± 6.9; 17.4 ± 5.8
Vaez et al. (2022)	Tehran, Iran	PCOS	QUR (31); Placebo (33)	QUR 500 mg/d, p.o	12 weeks	32.50 ± 5.59; 29.64 ± 5.87	23.5 ± 0.9; 25.3 ± 0.4	NM	NM	3.16 ± 1.43; 4.11 ± 1.56	4.07 ± 1.39; 3.87 ± 1.43	NM	NM

QUR, quercetin; PCOS, polycystic ovary syndrome; SD, standard deviation; BMI, body mass index; T, testosterone; SHBG, sex hormone-binding globulin; LH, luteinizing hormone; FSH, follicle-stimulating hormone; INS, insulin; HOMA-IR, homeostasis model assessment insulin resistance; NM, not mentioned; *, *p* < 0.05.

### 5.1 Effects of QUR on steroid hormones

HA is an independent risk factor for long-term health outcomes associated with PCOS (Legro et al., 2013), and the incidence of HA in PCOS patients is as high as 60%–80% (Ye et al., 2021). HA is an important clinical feature in patients with PCOS, which is often associated by increased T and LH. In women, androgen precursors are produced by the adrenal glands and then activated in the ovaries and peripheral tissues to testosterone (Rosenfield and Ehrmann, 2016). Excessive androgen production in the ovaries is considered the most important cause of PCOS, and the resulting excess estradiol leads to disturbance of the hypothalamic-pituitary-gonadal-adrenal axis (Rosenfield and Ehrmann, 2016). Increased gonadotropin-releasing hormone (GnRH) which derives from disorders of the axis leads to higher frequency luteinizing hormone (LH) pulsation, stimulation of LH-mediated androgen production and disruption of follicular development (Szeliga et al., 2022). Elevated levels of LH stimulate ovarian theca cells to secrete androgens.

Changes in LH and T levels are important parameters in patients’ treatment with QUR. There was a randomized placebo-controlled double-blind clinical trial of 84 women aged 20–40 years with a BMI of 25–40 kg/m^2^, randomized to QUR group and placebo group, QUR group treatment with 1 g QUR per day for 12 weeks. This study found that T levels (0.71 ng/dl in QUR vs. 0.77 ng/dl in placebo; *p* < 0.001) and LH levels (8.42 IU/L in QUR vs. 8.68IU/L in placebo; *p* = 0.009) were significantly lower in the QUR-treatment group than in the placebo group (Rezvan et al., 2017). Another study recruited 78 overweight or obese women (25 ≤ BMI ≤40 kg/m^2^, 20–40 years) with PCOS. Patients were randomized to receive 1,000 mg/day QUR or placebo for 12 weeks, then T (0.72 ± 0.15 vs. 0.76 ± 0.12 ng/ml) and LH (8.05 ± 2.88 vs. 8.77 ± 1.99 mIU/ml) levels were significantly lower in the QUR group compared with the placebo group (Khorshidi et al., 2018). A newsiest clinical study (Vaez et al., 2022) has found that, after 12 weeks of QUR treatment, there was no significant difference in BMI and hormone levels between the QUR and PCOS groups, but patients from the QUR group had lower LH than before treatment (4.35 ± 1.62 vs. 3.06 ± 1.43 mIU/ml; *p* = 0.029).

However, there is no relevant studies on the dose and duration of QUR, and the selection of the above-mentioned research subjects is relatively single, the sample size is small, and the detected hormones are relatively limited. Subsequently, more well-designed multi-center large samples are needed for further confirmation. Studies involving non-selective population would further validate the beneficial effects of QUR. Since in addition to T and LH, changes in FSH, anti-mullertubulin (AMH), insulin-like growth factor (IGF) and androgen invertase may all be reference indicators of oocytes damage (Palomba et al., 2017), future studies that can detect more sex-related hormones will be more significant for our analysis of the effects of QUR on steroid hormones.

### 5.2 Effects of QUR on insulin resistance and the metabolism of glycolipids

IR plays a significant important role in the development of PCOS (Amsterdam, 2012). About 50%–70% of PCOS patients have IR (Azziz et al., 2005), and up to 88% of women with PCOS are at risk of being overweight and obese (Barber et al., 2006; Lim et al., 2012). In IR state, insulin binding to its receptor is disrupted or the latter is ineffectively activated by insulin, forcing the pancreatic *ß*-cells to secrete excess insulin into the circulation to maintain euglycemia (Diamanti-Kandarakis and Dunaif; 2012). HA and IR may affect each other, leading to neuroendocrine disorders, reproductive failure and abnormal glycolipid metabolism (Wang et al., 2019). In addition, excess insulin and LH synergistically increase androgen release and inhibit the secretion of hepatic SHBG (Szeliga et al., 2022), creating a vicious circle between HA and IR. Whether HA causes IR or IR causes HA has not been elucidated yet. Currently, it is believed that the occurrence of PCOS is the result of the joint action of HA and IR.

As a potential treatment agent for PCOS, whether QUR could improve the IR status machine-related indicators of PCOS patients is also the focus of clinical attention. In addition to the changes in LH and T levels in the randomized controlled studies, the changes in total fasting serum adiponectin, high molecular weight (HMW) adiponectin, glucose, insulin levels, and HOMA index in patients with PCOS after QUR treatment were closely observed. It is found that there were significant inverse correlations between the fold changes of HOMA-IR and serum total adiponectin (*p* < 0.001) and high-molecular-weight adiponectin (*p* = 0.001), between the changes in adiponectin and BMI(*p* < 0.001) and weight change (*p* = 0.018) (Rezvan et al., 2017). A significant decrease has been observed in plasma concentration of resistin (−0.81 ± 0.45 *versus* 0.02 ± 0.49 ng/ml, *p* < 0.001) in QUR compared with the placebo group following supplementation in another study. In addition, resistin gene expression decreased 36% (0.64 ± 0.58 vs. 1 ± 0.56, *p* = 0.008) in QUR compared with the compare group. FBG (*p* < 0.001), insulin (*p* = 0.02), and HOMA-IR (*p* = 0.009) decreased significantly in the QUR group at the end of the study, despite there were no significant differences after adjusting for age, baseline waist circumference, BMI changes, and baseline values for each factor (Khorshidi et al., 2018).

To investigate the possible mechanism of QUR in improving IR in PCOS, the investigators conducted further studies. Previous studies have had clear evidence that adiponectin signaling is controlled by its receptors, adpR1 and adpR2, which partially regulate glucose and fatty acid metabolism through the activation of AMPK. And the adiponectin receptor is upregulated at the transcriptional and protein levels of PCOS female adipocytes, but not in peripheral blood mononuclear cells. QUR significantly upregulated the AdpR1 and AdpR2 transcript expression in isolated peripheral blood monocytes (1.32-and 1.46-fold respectively, *p* < 0.01). To further investigate the role of the adiponectin receptor signaling pathway, the researchers also analyzed the expression of its downstream molecular target, AMPK. The results showed that QUR addition increased the AMPK levels by 12.3% compared with the control group (Rezvan et al., 2018). QUR has been shown to induce adiponectin secretion in adipocytes *via* a peroxisome proliferator-activated receptor (PPAR) independent pathway (Wein et al., 2010). Whether this may also be the mechanism by which QUR improves IR in PCOS patients remains to be confirmed by further researches.

## 6 Effects of QUR TCM compound on PCOS

QUR exists in the plant root, flower, leaves, and fruit, such as apples, hawthorn, and root of Chinese herbal medicine, and some Chinese herbal medicine decoction may also contain QUR. However, due to the complex composition of Chinese herbal medicines, there is a lack of uniform evaluation criteria for the treatment of PCOS with Chinese herbal decoctions, and the clinical randomized controlled studies are very rarely. Therefore, the data on the therapeutic value of Chinese herbal medicines containing QUR for PCOS is relatively lacking.

A meta-analysis included five randomized controlled studies of 285 patients with PCOS and IR found that there was no statistically significant difference between the BUSHEN HUATAN group and the metformin group in improving LH, FSH, fasting blood glucose, and fasting insulin (Huang et al., 2020). Investigators applied network pharmacology and molecular docking methods to explore the possible mechanism of BUSHEN HUATAN in patients with PCOS. It revealed the role of BUSHEN HUATAN in improving IR and hormonal disorder in PCOS through multiple components, multi-target sites, and multiple routes, and found that QUR might be one of the core components to play more important value in the treatment of PCOS (Hong et al., 2014).

BUSHEN HUOXUE HUATAN decoction is also a classic prescription for treating PCOS (Ding et al., 2022). There was a non-randomized controlled study that enrolled 62 patients with PCOS and IR, and all patients received BUSHEN HUOXUE HUATAN decoction for 3 months. The ovulation rate, anthropometric measures, clinical symptoms, serological measures were measured and compared before and after treatment. It also investigated the possible mechanisms and targets of this decoction, network pharmacological analysis revealed that QUR is one of the most important active components of BUSHEN HUOXUE HUATAN decoction; while signal transducer and activator of transcription 3 (STAT3), Jun, AKT1, MAPK3, MAPK1, and TP53 are the most important drug targets. After 3 months of treatment, LH, L/FSH, T, DHEA, insulin and the area under the insulin curve were significantly reversed before treatment in PCOS patients. Serum inflammatory factors including human IL-18, IL-16, IL-1, IL-8, macrophage migration repressor, and human leukocyte differentiation antigen CD40 ligand levels were significantly reduced. And this study inferred that BUSHEN HUOXUE HUATAN decoction has potential therapeutic effects on PCOS, and the mechanism could be related to regulating hormone levels, improving IR, reducing inflammation, and promoting pregnancy. QUR may be an important active ingredient (Ding et al., 2022). There are similar studies, for example, YIJING decoction treatment for PCOS patients, which also considered QUR as one of the potentially effective components (Lin et al., 2022).

Unfortunately, there are no large sample randomized controlled studies to confirm the value of Chinese herbal medicines containing QUR in patients with PCOS. Because of the extremely complexity of its chemical composition, the specific mechanism of action and the role and value of QUR in treatment also need more studies to further confirm.

## 7 Conclusion and perspectives

PCOS is a common multifactorial endocrine disease, the main clinical features are ovarian enlargement or PCOM, HA, no ovulation or oligomenorrhea, accompanied by endocrine abnormalities, metabolic abnormalities, and reproductive dysfunction. Metabolic abnormalities such as IR and HA are considered to be the core links of PCOS. QUR is a natural antioxidant, anti-inflammatory, and anti-metabolizing flavonoid widely found in Chinese herbal medicines and fruits, and green leafy vegetables, as well as in many seeds. *In vitro* studies have shown that QUR can relieve oxidative stress and apoptosis of ovarian cells. Previous studies have found that QUR can effectively reduce serum testosterone, luteinizing hormone, the LH/FSH ratio, fasting blood glucose, fasting insulin, HOMA-IR, and blood lipids levels in PCOS patients or animal models of PCOS. Animal experiments have also shown that QUR can be a factor that increases the number of preantral, antral, and preovulatory follicles and corpora lutea counts, as well as decreasing atretic follicle count and eliminating the formation of cysts in the PCOS rats. Potential mechanisms of action may include anti-insulin resistance, anti-inflammatory resistance, anti-oxidative stress, anti-apoptosis, and downregulation of CNP/NPR2 expression.

At present, no appropriate cellular PCOS model has been reported, and the ovarian cell model needs to be explored to better simulate the PCOS disease environment. Studies have shown that QUR improves the sex hormone axis, however there is still debate over how QUR affects IR. To examine the effects of QUR on IR in PCOS animals from the viewpoints of various intervention durations and QUR dosages, more animal studies are required. Further research is necessary because discoveries about reproductive capacity of PCOS animal models are rare. To confirm the precise function of QUR in the treatment of PCOS patients, more extensive, high-quality prospective trials are needed, particularly those concentrating on QUR’s impact on IR status, HA status, and PCOS-related reproductive abnormalities. In the future, there will be more clinical trials applying QUR in PCOS patients.

## Glossary

ALT: alanine aminotransferase
AMPK: Amp-activated protein kinase
AOD: average optical density
AR: androgen receptor
AST: aspartate aminotransferase
adpR1: adiponectin receptor one
adpR2: adiponectin receptor two
Bax: Blc-2 Associated X protein
Bcl-2: B-cell lymphoma-2
BMI: body mass index
CAM: Complementary and alternative medicine
CAT: catalase
CHOP: C/EBP-homologous protein
CNP: C-type natriuretic peptide
CRP: C-reactive protein
CYP17A1: cytochrome P450 family 17 subfamily A member one
cAMP: cyclic adenosine monophosphate
cGMP: cyclic guanosine monophosphate
DALY: Disability Adjusted Life Year
DHEA: dehydroepiandrosterone
DPP-4: dipeptidyl peptidase-4
DPPH: 2,2-Diphenyl-1-picrylhydrazyl
E2: estradiol
ERα: estrogen receptor α
FBG: fasting blood glucose
FIS: fasting insulin
FSH: follicule-stimulating hormone
GK: glucokinase
GLP-1R: glucagon-like peptide-1 receptor
GLUT4: glucose transporters
GnRH: Gonadotropin-releasing hormone
GPx: Glutathione peroxidase
GSH: glutathione
GSTL: glutathione-S-transferase
HA: hyperandrogenism or hyperandrogenemia
HDL-C: high density lipoprotein-cholesterol
HK: hexokinase
HMW: high molecular weight
HOMA: Homeostasismodel Assessment
IGF-1: insulin-like growth factors -1
IGF2R: insulin like growth factor 2 receptor
IL-1β: leukocyte factor 1
IL-6: leukocyte factor 6
IL-18: leukocyte factor 18
IR: insulin resistance
IRS-1: insulin receptor substrate-1
LDH: lactate dehydrogenase
LDL-C: low density lipoprotein-cholesterol
LH: luteinizing hormone
INS: insulin
MCP-1: monocyte chemotaxis protein-1
MDA: malondialdehyde
MIP-lα: macrophage inflammation protein-lα
mRNA: messenger RNA
NF-κB: Nuclear Factor Kappa B
NM: not mentioned
NO: nitric oxide
NPR2: natriuretic peptide receptor two
Nrf2: NF-E2-related factor 2
OCT-4: Octamer-binding transcription factor 4
OD: ovulatory dysfunction
OX-LDL: oxidized low density lipoprotein
PCNA: proliferating cell nuclear antigen
PCOM: polycystic ovary morphology
PCOS: Polycystic ovary syndrome
PDE3A: Phosphodiesterase 3 A
PERK: protein kinase R-like ER kinase
PI3K: Phosphatidylinositide 3-kinases
PPAR: peroxisome proliferator-activated receptor
p.o.: peros
QUR: quercetin
ROS: reactive oxygen species
SD: Sprague–Dawley, Standard deviation
SEM: standard error of mean
SGLT2: sodium-glucose cotransporter
SHBG: sex hormone binding globulin
SIRT-1: Sirtuin one
SOD: superoxide dismutase
STAT3: signal transducer and activator of transcription 3
s.c.: subcutaneous injection
T: testosterone
TAC: total antioxidant capacity
TC: cholesterol
TG: triglycerides
TLR-4: Toll-like receptor 4
TNF-α: tumor necrosis factor α
Trx: thioredoxin
TZD: thiazolidinediones
VEGF: vascular endothelial growth factor
WBC: white blood cell count
XIAP: X-linked inhibitor of apoptosis protein
8-IP: 8-isoprostantin
